# Finding suitable candidates for vacuum bell therapy in pectus excavatum patients

**DOI:** 10.1038/s41598-021-02250-x

**Published:** 2021-11-23

**Authors:** Eunjue Yi, Kwanghyoung Lee, Younggi Jung, Jae Ho Chung, Han Sung Kim, Sungho Lee, Hyonggin Ahn

**Affiliations:** 1 Department of Thoracic and Cardiovascular Surgery, Korea University Anam Hospital, 73, Koryeodae-ro, Seongbuk-gu, Seoul, 02841 Republic of Korea; 2 Department of Biomedical Engineering, College of Software and Digital Healthcare Convergence, Yonsei University, Wonju, Republic of Korea; 3 Department of Biostatistitcs, Korea University College of Medicine, 73, Koryeodae-ro, Seongbuk-gu, Seoul, 02841 Republic of Korea

**Keywords:** Diseases, Medical research

## Abstract

Vacuum bell therapy has been acceptable substitute for pectus excavatum patients who want to improve their appearance but avoid surgical correction. The aim of this study was to assess the pre-treatment characteristics of patients with pectus excavatum and to establish characteristics that can potentially help identify ideal candidates for vacuum bell therapy. Expected improvements in thoracic indices were evaluated using pre-treatment chest computed tomography, which was performed before and after applying a vacuum bell device. Treatment results after 1-year of application were evaluated using changes in the Haller index before and after treatment. The patients were categorized into two groups according the post- treatment changes in Haller index calculated using chest radiographs: those with changes in Haller index less than 0.5 (Group 1) and those with greater than or equal to 0.5 (Group 2). Pre-treatment Haller index was significantly lower in Group 1 than in Group 2 (3.1 ± 0.46 vs. 4.2 ± 1.14, respectively, *p* < 0.001). The expected improvement in Haller index in Group 2 was significantly higher than that in Group 1 (3.3 ± 0.60 vs. 2.8 ± 0.54, respectively, *p* = 0.001). The cut-off value of the expected improvement in Haller index was 0.46 with a sensitivity of 75.8% and a specificity of 83.3%. Patients who demonstrated pliability with a vacuum bell were identified as suitable candidates.

## Introduction

Although minimally invasive surgical repair (MIRPE) is the gold standard treatment for pectus excavatum (PE)^1^, there is a need for less invasive therapeutic methods that can effectively manage pain and are not associated with severe complications^2^. The vacuum bell (VB) device has been used for several decades as a feasible and safe treatment for patient with PE^3^. It has demonstrated effectiveness in carefully selected patients with PE who wish to avoid surgical corrections^3,4^. However, it is contraindicated in conditions, such as musculoskeletal disorder, vasculopathy, coagulopathy, and cardiac disorders^4^.

In this study, we assessed our clinical experiences of vacuum bell therapy, evaluated the pre-treatment characteristics and treatment outcomes using various indices, and tried to identify appropriate clinical traits for successful vacuum bell devices, therefore suggest more favorable treatment strategy for each patient.

## Materials and methods

### Study approval

This study protocol and a waiver of informed consent were approved by the Institutional Review Board of Korea University Anam Hospital (IRB number: 2018AN0080). All methods including retrospective data collection and analysis were performed in accordance with relevant Korean guidelines and regulations.

### Patients

Between January 2016 and December 2019, we encountered 119 patients with PE who received vacuum bell therapy for more than 1 year. Vacuum bell devices were supplied by Eckart Klobe (https://eckartklobe.com, Mannheim, Germany) and the application was approved by Korean Ministry of Food and Drug Safety (Product-License No.; 16-4114, U. S. Food and Drug Administration regulation number; 890.3490). Of them, 26 patients decided to stop the treatment and underwent surgical correction during the follow-up period. They were excluded from the study. Patients who did not undergo chest computed tomography (CT) before undergoing vacuum bell (VB) therapy (30 patients) were also excluded.

Our inclusion criteria were as follows: (1) received VB therapy for more than 1 year; (2) visited the outpatient department at least four times to evaluate treatment effects; and (3) underwent chest CT before starting treatment.

The medical records of the patients were reviewed retrospectively. Pre-treatment characteristics, including Haller index (HI), and post-treatment results were evaluated. The changes in HI after VB application were calculated using chest radiographs. The patients were classified into two groups according to the post-treatment changes in HI of < 0.5 (Group 1) or ≥ 0.5 (Group 2). This study was approved by the Institutional Review Board of the Korea University Anam Hospital (IRB number: 2018AN0080).

### Definition of thoracic indices

**Figure 1:**
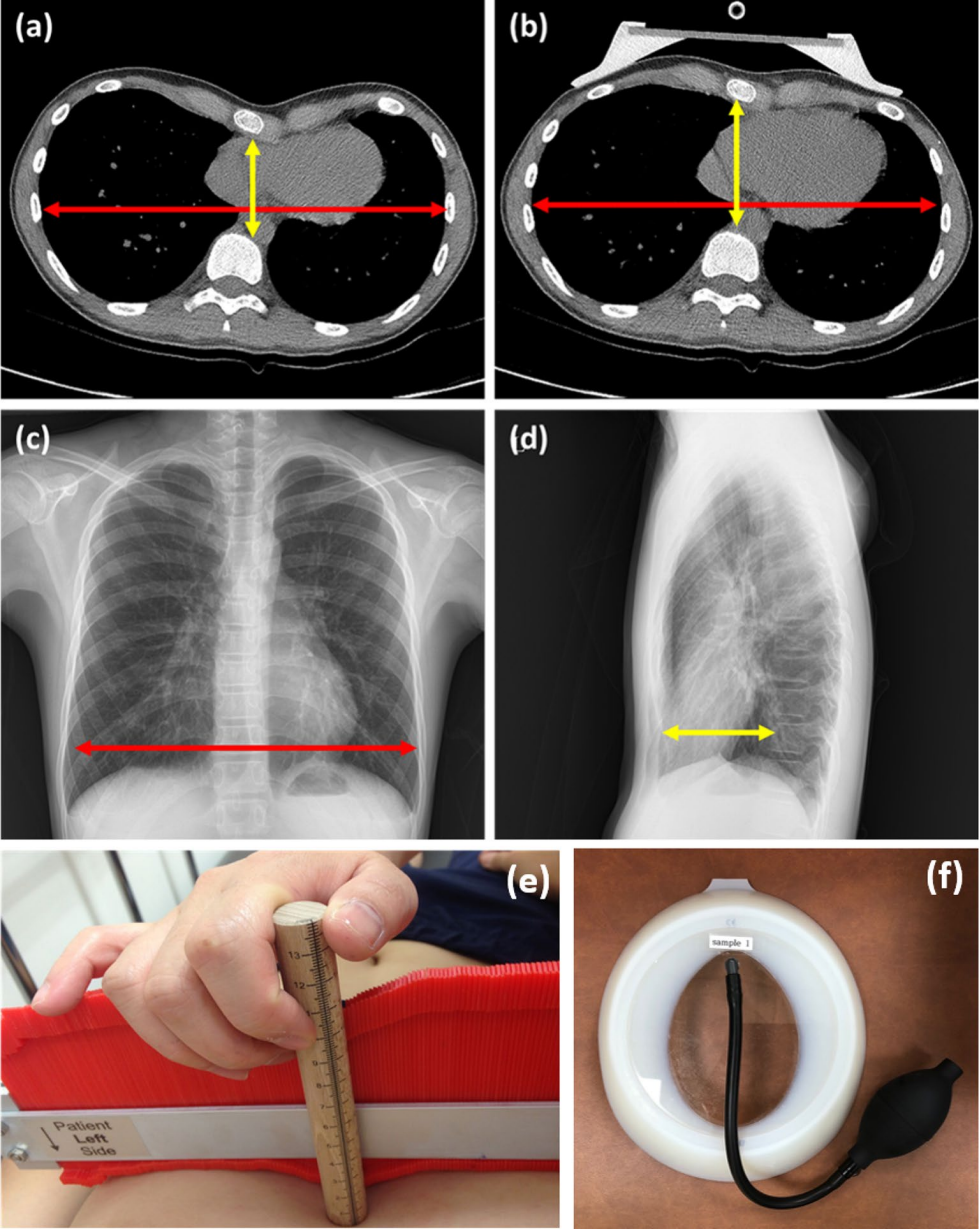
Evaluation of pre-treatment and expected improvements in thoracic indices. (**a**) Chest computed tomography (CT) taken before vacuum bell application is shown. Haller index was derived by dividing the largest horizontal diameter inside the ribcage (red arrow) by the anteroposterior diameter (shortest distance between the vertebrae and sternum, yellow arrow). (**b**) Chest CT taken after vacuum bell application is shown. Expected improvement in Haller index was measured as described above. (**c**) Posterior-anterior (PA) view on chest radiograph is illustrated. The greatest transverse diameter (largest horizontal diameter within the ribcage) was measured (red arrow) (**d**) Lateral view on chest radiograph is shown. The anteroposterior diameter (shortest distance between vertebrae and sternum) was measured (yellow arrow). Haller Index was calculated by dividing the transverse diameter by anteroposterior diameter. (**e**) Measurement of depth of PE. The anteroposterior distance of the deepest point of the sternum, was measured with the patient in a horizontal supine position on a flat table during deep inhalation using a designed scale rod. (**f**) Vacuum bell device (largest one). They were supplied by Eckart Klobe (https://eckartklobe.com, Mannheim, Germany).

The thoracic indices measured using chest CT and chest radiographs were as follows. Haller index was calculated according to the definition by Haller in 1987; an index derived from dividing the greater transverse diameter of the chest by the shortest anteroposterior diameter using chest CT images^5^. Chest radiographs also could be used for calcalation^6^. On chest radiographs, HI was calculated by dividing the greater transverse diameter on posteroanterior view by the shortest distance between the vertebrae and sternum (shortest anteroposterior diameter) on lateral view (Fig. 1).

**Figure 2:**
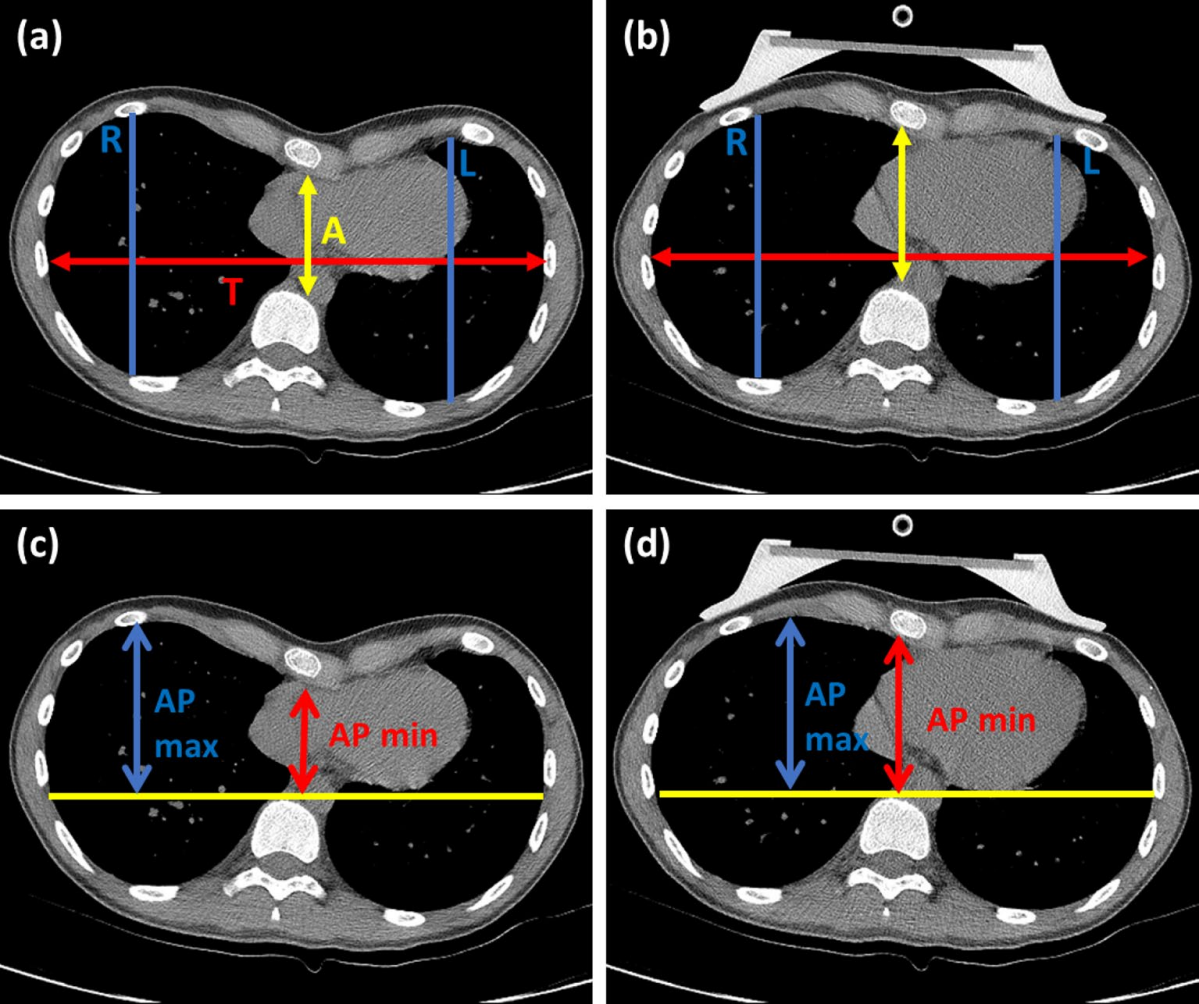
Measurement of asymmetry index (AI) and correction index (CI) using chest computed tomography (CT). (**a**) Measuring AI before applying vacuum bell (VB) device: The largest sagittal distance of the right (R) and left (L) chest at the same slice on chest CT as that used for HI was used in the formula was AI = R/L, where T is the greater transverse diameter and A is the shortest distance between the vertebrae and sternum. AI > 1.0 implies right asymmetry, while AI < 1.0 indicates left-skewed PE (**b**) Measurement of AI during application of a VB device: Assessments were performed using the chest CT slice as that used before. (**c**) Measuring CI before applying a VB device: The minimum distance between the posterior mediastinum and anterior spine (AP min) and maximum distance between the anterior spine and anterior portion of chest wall (AP max) were measured. CI was calculated as follows: CI = ([AP max]-[Ap min])/[AP max]).

The asymmetry index (AI) designed to quantify chest deformities that could not be recognized using HI^7^ included the largest sagittal distance of the right and left chest within the same slice of chest CT as that used for HI measurement. The correction index (CI), a novel index proposed by S.D. St. Peter^8^, was calculated using chest CT and expressed as a percentage ratio. It has been reported to be related to the possible percentage of correction in chest deformities using MIRPE^8,9^. Calculation formula were described in Fig. 2.

Post-treatment Haller indices were calculated using chest radiographs taken 1-year after starting treatment. They were measured on similar slices and were used for post-treatment comparison with the pre-treatment images.

### Pre-treatment examination

A specially designed non-enhanced chest CT for patients with PE was performed before deciding the treatment method. Chest CT was performed to obtain two sets of non-enhanced transverse images. One was taken during the ordinary condition, and the other during application of a vacuum bell device on the chest wall defect. Patients were instructed to sustain inhalation status while Chest CT or chest radiography was taken. Chest radiography and electrocardiography were routinely performed before starting the treatment.

After careful physical examination and review of imaging studies, patients were informed about the conservative treatment and possibility of surgery. For patients who wanted to try the non-invasive treatment, the first application of a vacuum bell device was performed in an outpatient department based on precise instructions and under careful supervision of attending clinicians.

### Treatment protocols

Basic vacuum bell protocols included 30-min application twice a day. Patients were encouraged to extent duration or increase the number of times. Duration could be last up to several hours (usually two to four hours) if they were tolerable and no complications such as skin irrigation was observed. Presence of a caregiver was compulsory if the patients were under 18, and they were encouraged to learn how to use the device if the patients were under 15 years of age.

Four types of vacuum bell devices (16, 19, and 26 cm, and a specialized dumbbell-shaped device for women) with different sizes and shapes were available. The appropriate size and shape of the devices were carefully chosen after several trials.

### Measurement of pre-treatment thoracic indices

Thoracic indices including HI, AI, and CI were measured in two ways using chest CT scans which had two phases, one without and the other with VB devices (Table 2). The largest transverse and anteroposterior diameters at the deepest points of the sternum were measured with and without the application of the vacuum bell device on chest CT. The improvements in thoracic indices were measured and compared between the two phases. The pre-treatment HI was also measured using chest roentgenography to compare with the post-treatment outcomes.

The range of sternal depression (depth of PE), which is the anteroposterior distance of the deepest point of the sternum, was measured with the patient in a horizontal supine position on a flat table during deep inhalation using a designed scale rod. This clinical index was evaluated twice during the first visit. It was measured without any manipulation. Then the vacuum bell was applied on the patient’s chest for 30 min. Second measure was performed after waiting 5 min from the time of the device had been removed. The expected sternal elevation was calculated using these two parameters.

### Follow-up

**Figure 3:**
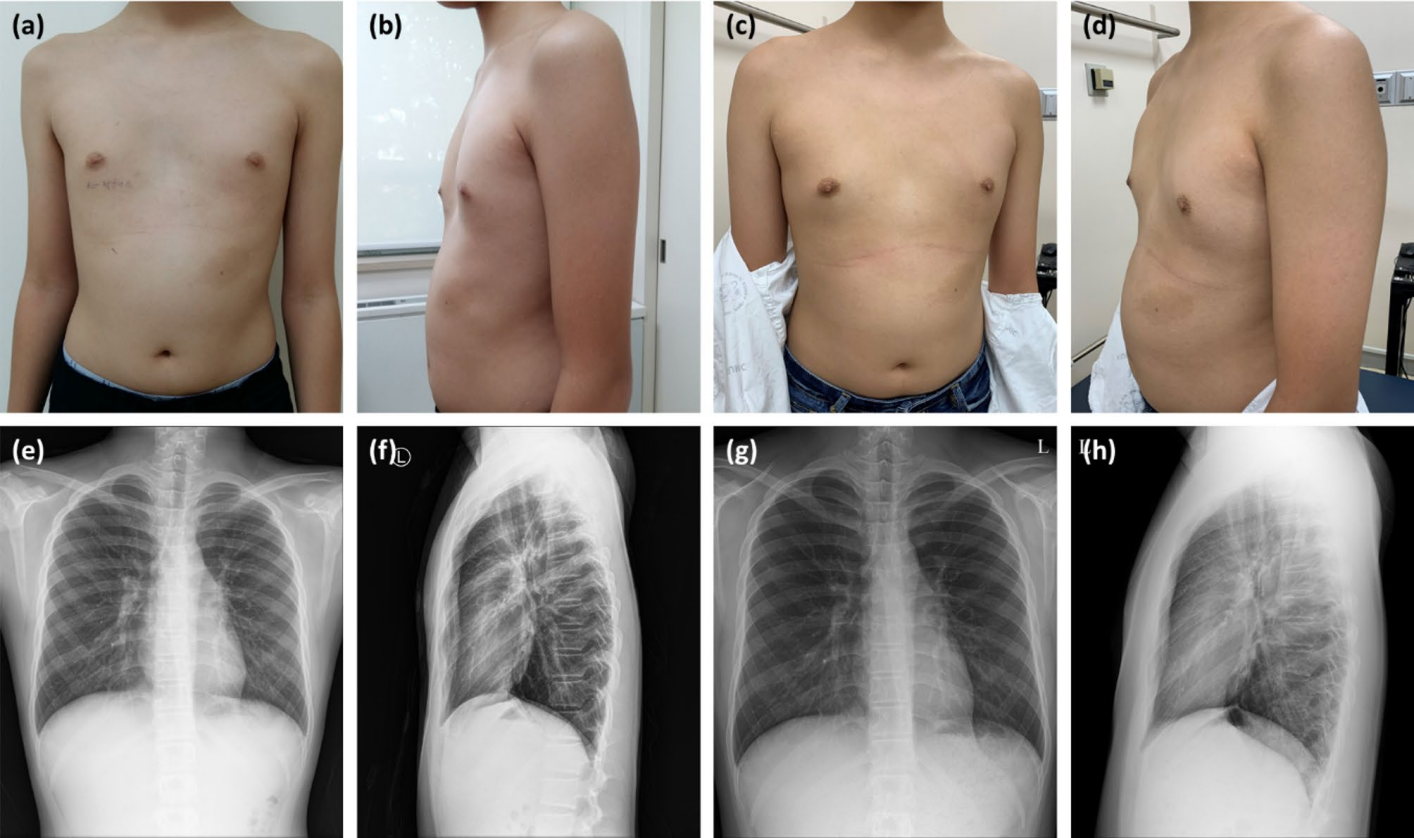
Evaluation of treatment outcomes after 1 year of vacuum bell (VB) application. Pictures and chest radiographs were taken from a 17-year-old male patient just before starting treatment (**a**, **b**, **e**, **f**) and at 1 year after VB therapy (**c**, **d**, **g**, and **h**). (**a**) Anterior view before VB application; (**b**) lateral view before VB application; (**c**) anterior view at 1 year after treatment; (**d**) lateral view at 1 year after treatment; (**e**) anteroposterior view on chest radiographs taken before starting therapy; (**f**) lateral view before starting therapy; (**g**) anterolateral view after 1 year of treatment; and (**h**) Lateral view after 1 year of treatment.

Regular outpatient follow-ups were recommended every 3 months. The depth of PE was measured at each visit, and chest radiography was performed after 1 year of treatment. Photographic documentation was obtained at the first and 1-year follow-up visits (Fig. 3). Follow-up chest CT was not performed to avoid unnecessary exposure to radiation. Duration and times of VB wearing were reported from patients or their caregivers whenever they visited. Non-compliance were defined when the patients or caregivers had reported that they did not wear the device at least one time per day. Presence of complications including chest tightness and skin erosion were also dependent on patients’ statement. Skin erythema was confirmed by a physician who had observed the patient.

### Estimation of treatment efficacy

For estimation of the treatment response, changes in HI were calculated using the initial and 1-year follow-up chest radiographs. The initial HI on chest CT and chest radiography were positively correlated (Pearson coefficient = 0.905, *p* < 0.001). A reduction of ≥ 0.5 in HI (since the mean overall HI before the treatment was 3.7 and 3.25 was considered the surgical threshold) was considered a successful treatment outcome in our study. The daily duration and the frequency of the vacuum bell device was recorded as reported by the patients and caregivers. Subsequently, the compliance and complications were assessed.

### Evaluation of pre-treatment variables for identifying suitable candidates

The patients were categorized into two groups: Group 1 included patients with changes in HI < 0.5, and Group 2 included those with changes in HI ≥ 0.5. Pre-treatment characteristics, including expected improvements in thoracic indices and sternum depth, were compared. The cut-off value for the expected improvement in HI in Group 2 was measured.

### Statistical analysis

Chi-square and Fisher’s exact tests (when expected value of data was lower than 5) were used for statistical comparison for categorical variables. Student’s t-test for parametric and Mann–Whitney tests for nonparametric variables were applied to compare continuous variables. The Receiver Operator Curve (ROC) analysis was performed and cut-off value was calculated. Significant differences were defined when the p value was less than 0.05 with 5% significance level. Statistical analyses were performed using SPSS 20.0 (SPSS Inc., Armonk, NY, USA).

## Results

### Patient demographics and initial examinations

A total of 63 patients were enrolled in this study. Group 1 comprised 33 patients (31 men and two women), and Group 2 consisted of 30 patients (all men). The body mass index (BMI) in Group 2 was significantly lower than that in Group 1 (*p* = 0.001), while other demographic factors demonstrated no significant differences. The pre-treatment patient characteristics and clinical information are summarized in Table 1.

**Table 1:** Patient characteristics.

Variables	Group 1 (N = 33)		Group 2 (N = 30)		Total		*p*-value
	mean ± sd	(95% CI)	mean ± sd	(95% CI)	mean ± sd	(95% CI)	
Age	16 ± 7.54	(845)	(14.2 ± 4.18)	(9–34)	15.4 ± 6.23	(8–45)	0.254
**Gender**							0.493
Female	2 (6.1%)		0 (0.0%)		2 (3.2%)		
Male	31 (93.9%)		30 (100.0%)		61 (96.8%)		
BMI (kg/m^2^)	19.0 ± 2.39	(16–25)	16.4 ± 3.84	(9–21)	(17.8 ± 3.45)	(9–25)	0.001
**Family history**							0.094
No	32 (56.1%)		25 (83.3%)		57 (90.5%)		
Yes	1 (3.0%)		5 (16.7%)		6 (9.5%)		
**Smoking history**							0.334
None	29 (87.9%)		29 (96.7%)		58 (92.1%)		
Ex	2 (6.1%)		0 (0.0%)		2 (3.2%)		
Current	2 (6.1%)		1 (3.3%)		3 (4.8%)		
**Comorbidity**							0.535
Mitral regurgitation	0 (0.0%)		1 (3.0%)		1 (1.6%)		
Scoliosis	1 (3.0%)		1 (3.0%)		2 (3.2%)		
Arrythmia	1 (3.0%)		0 (0.0%)		1 (1.6%)		
Atopy	0 (0.0%)		1 (3.0%)		1 (1.6%)		
**Onset periods**							0.922
Infant (0–1-year)	0 (0.0%)		1 (3.3%)		1 (1.6%)		
Toddler (1–3-year)	1 (3.0%)		1 (3.3%)		2 (3.2%)		
Child (3–10-year)	9 (27.3%)		8 (26.7%)		17 (27.0%)		
Preadolescent (10–13-year)	13 (39.4%)		11 (36.7%)		24 (38.1%)		
Adolescent (13–19-year)	8 (24.2%)		8 (26.7%)		16 (25.4%)		
Adult (> 19-year)	2 (6.1%)		1 (3.3%)		3 (4.8%)		
**EKG findings**							0.473
NSR	31 (93.9%)		26 (86.7%)		57 (90.5%)		
Sinus bradycardia	2 (6.1%)		3 (10.0%)		5 (7.9%)		
Incomplete RBBB	0 (0.0%)		1 (3.3%)		1 (1.6%)		
**Nuss operation history**							0.321
Yes	0 (0.0%)		1 (3.3%)		1 (1.6%)		
No	33 (100.0%)		29 (96.6%)		62 (98.4%)		
**Symptoms**							0.359
Chest discomfort	0 (0.0%)		1 (3.3%)		1 (1.6%)		
Palpitation	0 (0.0%)		1 (3.3%)		1 (1.6%)		
Dyspnea	1 (3.0%)		0 (0.0%)		1 (1.6%)		
Cough	0 (0.0%)		1 (3.3%)		1 (1.6%)		
Low body weight	0 (0.0%)		1 (3.3%)		1 (1.6%)		
None	32 (97.0%)		26 (86.7%)		58 (92.1%)		

*BMI* body mass index, *EKG* electrocardiography, *RBBB* right bundle branch block, *NSR* Normal sinus rhythm, *sd* standard deviation.

### Comparison of pre-treatment thoracic indices with post-treatment values

Both pre-treatment HI and depth of PE in Group 2 were significantly higher than those in Group 1 (*p* < 0.001 and *p* = 0.021, respectively). The expected improvement in HI was significantly higher in Group 2 than that in Group 1 (*p* = 0.001), which appeared to be correlated with post-treatment changes in HI after 1 year (0.93 ± 0.400 vs. 0.18 ± 0.197, respectively, *p* < 0.001); however, the response with the depth of PE was not. The expected depth of PE (Changes in AP diameter with and without VBT application) was significantly better in Group 2 than in Group 1 (9.3 ± 5.48 vs. 15.0 ± 6.80, respectively, *p* = 0.001) but post-treatment response demonstrated no statistically significant differences (0.66 ± 0.838 vs. 0.67 ± 1.002, respectively, *p* = 0.957). The pre-treatment and post-treatment outcomes are summarized in Table 2.

**Table 2:** Thoracic indices before and after vacuum bell therapy.

Variables	Group 1 (N = 33)			Group 2 (N = 30)			Total (N = 63)			*p*-value
	mean ± sd	(95% CI)		mean ± sd	(95% CI)		mean ± sd	(95% CI)		
**Haller index**										
*Pre-treatment*										
CXR	3.1 ± 0.46	(2.2–8.3)		4.2 ± 1.14	(3.0–8.3)		3.6 ± 1.00	(2.2–8.3)		< 0.001
**Chest CT**										
Without VB application	3.2 ± 0.79	(2.2–6.7)		4.2 ± 1.16	(2.9–8.5)		3.7 ± 1.10	(2.2–8.9)		< 0.001
With VB application	2.8 ± 0.54	(2.1–5.2)		3.3 ± 0.60	(2.2–4.5)		3.0 ± 0.61	(2.1–5.2)		0.001
**(Expected HI)**										
Changes in AP diameter (mm)	9.3 ± 5.48	(1.2–21.7)		15.0 ± 6.80	(2.4–28.2)		12.0 ± 6.72	(1.2–28.2)		0.001
Changes in AP diameter (%)	12.7 ± 8.23	(9.8–15.6)		27.6 ± 19.82	(20.1–35.0)		19.8 ± 16.57	(15.6–23.9)		< 0.001
**Post-treatment (CXR)**										
1-year FU	2.9 ± 0.46	(2.0–3.9)		3.2 ± 0.93	(2.4–6.8)		3.1 ± 0.73	(2.0–6.8)		0.292
**Asymmetry index**										
Without VB application	1.00 ± 0.780	(0.84–1.21)		0.97 ± 0.638	(0.85–1.12)		0.98 ± 0.719	(0.84–1.21)		0.366
With VB application (Expected)	1.00 ± 0.061	(0.89–1.11)		0.97 ± 0.079	(0.82–1.15)		0.98 ± 0.071	(0.82–1.15)		0.228
**Correction index**										
Without VB application	0.14 ± 0.826	(0.00–0.38)		0.23 ± 0.125	(0.03–0.49)		0.18 ± 0.114	(0.00–0.49)		0.003
With VB application (Expected)	0.06 ± 0.061	(0.00–0.23)		0.15 ± 0.125	(0.00–0.59)		0.10 ± 0.106	(0.00–0.59)		0.001
**Changes in HI**										
Pre-post VB application	0.39 ± 0.308	(0.02–1.53)		0.97 ± 0.782	(0.28–4.37)		0.67 ± 0.648	(0.02–4.37)		< 0.001
After treatment	0.18 ± 0.197	(0.25–0.46)		0.93 ± 0.400	(0.50–2.08)		0.54 ± 0.487	(0.25–2.08)		< 0.001
**Depth of PE (cm)**										
Initial	2.3 ± 1.14	(0.0–6.2)		2.7 ± 0.92	(0.0–4.3)		2.4 ± 1.06	(0.0–6.2)		0.021
5-min After VB application	1.9 ± 0.80	(0.0–3.7)		2.2 ± 0.89	(0.0–3.7)		2.0 ± 0.85	(0.0–3.7)		0.088
3-month FU	2.0 ± 0.76	(0.0–3.5)		2.3 ± 0.84	(0.0–4.0)		2.2 ± 0.81	(0.0–4.0)		0.030
6-month FU	1.9 ± 0.82	(0.0–3.4)		2.2 ± 0.80	(0.0–3.5)		2.0 ± 0.83	(0.0–3.5)		0.053
1-year FU	1.6 ± 0.92	(0.0–3.2)		2.0 ± 0.81	(0.0–3.2)		1.8 ± 0.88	(0.0–3.2)		0.061
Changes after treatment	0.67 ± 1.002	(− 1.50–3.10)		0.66 ± 0.838	(− 2.40–2.50)		0.67 ± 0.921	(− 2.40–3.10)		0.957
1-year changes after treatment (%)	32.6 ± 33.44	(19.9–45.3)		27.7 ± 23.05	(18.8–36.7)		30.2 ± 28.66	(22.6–37.8)		0.665
Follow-up BMI	19.5 ± 2.25	(18.6–20.5)		18.0 ± 5.46	(15.9–20.0)		18.7 ± 4.29	(17.6–19.9)		0.390
Poor compliance	6 (18.2%)			5 (16.7%)			11 (17.5%)			1.000
**Vacuum bell application duration (Hour)**										0.669
0.5	3 (9.1%)			4 (13.3%)			7 (11.1%)			
1	25 (75.8%)			20 (66.7%)			45 (71.4%)			
2	5 (15.2%)			5 (16.7%)			10 (15.9%)			
3	0 (0.0%)			1 (3.3%)			1 (1.6%)			
**Vacuum bell application frequency (per day)**										0.671
1	11 (33.3%)			12 (40.0%)			23 (36.5%)			
2	17 (51.5%)			13 (43.3%)			30 (47.6%)			
3	4 (12.1%)			5 (16.7%)			9 (14.3%)			
4	1 (3.0%)			0 (0.0%)			1 (1.6%)			
**Vacuum bell size**										0.487
1	5 (15.2%)			7 (23.3%)			12 (19.0%)			
2	22 (52.4%)			20 (66.7%)			42 (66.7%)			
3	4 (57.1%)			3 (10.0%)			7 (11.1%)			
4	6 (6.1%)			0 (0.0%)			2 (3.2%)			
**Complications**										0.457
Chest tightness	1 (3.0%)			1 (3.3%)			2 (3.2%)			
Skin erosion	1 (3.0%)			2 (6.7%)			3 (4.8%)			
Skin erythema	0 (0.0%)			1 (3.3%)			1 (1.6%)			

Haller indices were calculated both from pretreatment imaging modality including pretreatment chest CT or chest X-ray as well as chest X-ray after 1-year treatment. Asymmetry and Correction indices were measured only from pretreatment chest CT scans. Expected indices were estimated under VB application in chest CT and could imply possible changes when the treatment would proceed.

*CT* computed tomography, *CXR* chest radiography, *FU* follow-up, *HI* Haller index, *PE* pectus excavatum, *sd* standard deviation.

### Cut-off values based on pre-treatment estimation

**Figure 4:**
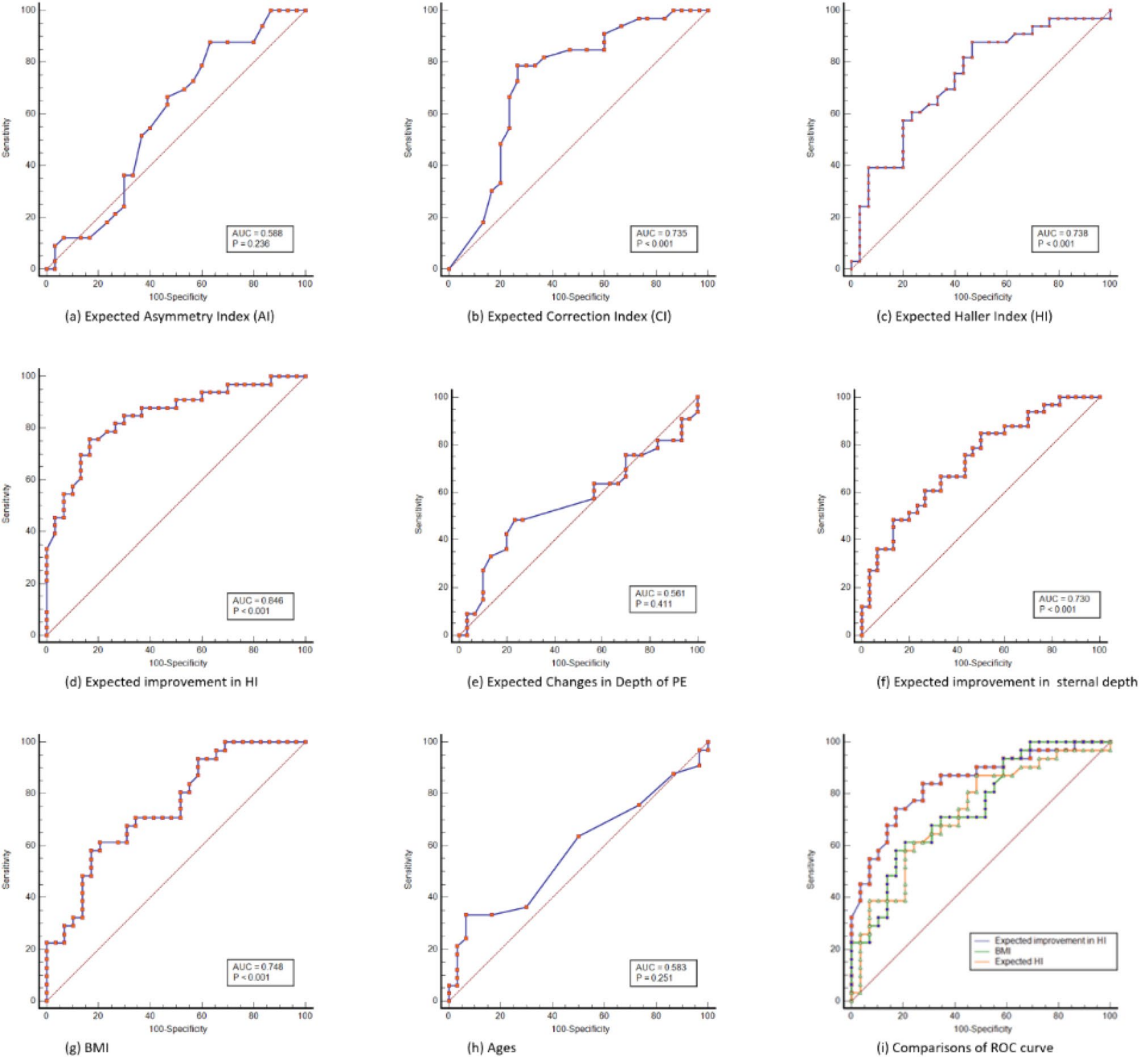
ROC curves of pretreatment variables. (**a**)Expected Asymmetric index. The cut-off value was 0.92 with 87.8% sensitivity and 36.7% specificity, which showed no statistical significance (*p* = 0.236). (**b**) Expected Correction index. The cut-off value was 0.07, with 78.8% sensitivity and 73.3% specificity (*p* < 0.001). (**c**) Expected Haller index. The cut-off value was 3.1, with 87.9% sensitivity and 53.3% specificity (*p* < 0.001). (**d**) Expected improvement in Haller index. The cut-off value was 0.46, with 75.8% sensitivity and 83.3% specificity (*p* = 0.001) (**e**) Expected improvement in depth of PE. The cut-off value was 0.4 cm, with 48.5% sensitivity and 76.7% specificity which showed no statistical significance (*p* = 0.411). (**f**) Expected improvement in sternal depth (calculated from chest CT). The cut-off value was 0.8 cm, with 48.5% sensitivity and 86.7% (*p* < 0.001). (**g**) BMI. The cut-off value was 18.5, with 58.0% sensitivity and 82.8% (*p* < 0.001). (**h**) Age. The cut-off value was 16, with 33.0% sensitivity and 93.3%, which showed no statistical significance (*p* = 0.251). (**i**) comparison with ROC curves of variables which showed higher classification performance. The Expected improvement in HI revealed better capability than other variables.

The estimated cut-off value in Group 2 in expected improvement in HI a receiver-operating characteristic (ROC) curve of 0.46, sensitivity of 75.7%, and specificity of 83.3% (area under the curve = 0.846, *p* < 0.001). The ROC curve is shown in Fig. 4.

## Discussion

Conservative management for PE using a suction device had suffered long periods of neglect before the efforts of E. Klobe in 1992 because of inadequate materials and relevant side effects^4^. The newly designed vacuum bell consisted of a silicon ring with a transparent polycarbonate window instead of glasses which old style suction cups were made of^10^.

Vacuum bell therapy has been successfully established as a conservative management for PE; however, there is a lack of information on precise clinical indications as well as assessment tools in estimating the treatment achievement, which limits its use by clinicians^11^. Difficulties in clinical evaluations may be attributed to the application of vacuum bell therapy tend to be largely dependent on a patient’s preference.

A prerequisite for vacuum bell therapy is a patient's desire for non-surgical corrections, despites not all patients could be beneficial from the non-invasive procedures. Reported clinical evidences relating to beneficial patient characteristics included a mild degree of PE, symmetric deformities, sternal depth greater than 1.5 cm, or younger age than preadolescence^12^. Clinicians cannot ignore the patients’ desire to avoid pain and possible complications caused by surgery even when their clinical experience suggests surgical solutions.

Certain patients abandoned this non-invasive but time-consuming indirect correction treatment and chose to undergo surgery after several months of disappointing treatment results. There may exist patients who would be contented when surgeries took priorities in their treatment strategies. However, application of vacuum device tended to be driven by commitment and endeavor from patients frequently, clinicians should prepare practical evidences for enhancing reasonable treatment choice.

The flexibility of the chest, and therefore the successful treatment (defined as improvement in HI ≥ 0.5 1-year after treatment) outcomes can be inferred from the expected improvement in HI (calculated from pre-treatment chest CT with or without VB devices) > 0.46, which showed most favorable discriminatory ability for successful treatment among other variables (AUC = 0.846 with *p* = 0.001, cut-off value of 0.46 with sensitivity of 75.7%, and specificity of 83.3%) in ROC curve analysis (Fig. 4).

This observation appears to be associated with chest wall flexibility, which has been reported to be related to successful treatment of PE, either surgically or non-surgically^13^. Patients with more flexible chest wall can be expected to achieve better treatment outcomes. Based on this factor, clinicians would be able to tell their patients how their chest deformities will change after 1 year of vacuum bell therapy. The expected CI or HI calculated using chest CT taken after applying a vacuum bell device and the expected improvement in sternal depth could be supplementary tools in predicting outcomes.

Unlike the other two thoracic indices associated with good treatment outcomes, AI was not correlated with the treatment response. Symmetry has been demonstrated to be related to improved therapeutic efficacy^12^. Although we did not examine the effects of symmetry on vacuum bell therapy, we believed that the discordance between improvement in chest deformity (improvement in HI ≥ 0.5) and expected AI would be reflected in the results.

Lower initial BMI was associated with better outcomes after VB therapy (*p* = 0.001), while other pre-treatment factors including gender and age showed no relevance. Although lower BMI had reported to be related with short-term efficacy of sternal lifting, probably lasting tens of minutes^14^, we did not find any evidences supporting it improved the depth of PE. We found that the initial BMI showed negative correlation with changes in AP diameter in chest CT (Pearson’s correlation coefficient (r); − 0.329, *p* = 0.010), however, not with changes of depth of PE (r = 0.089, *p* = 0.497). If the definition of successful VB treatment should contain significant improvement in depth of PE, lower initial BMI could not be considered as positive response factor for better outcomes.

**Figure 5:**
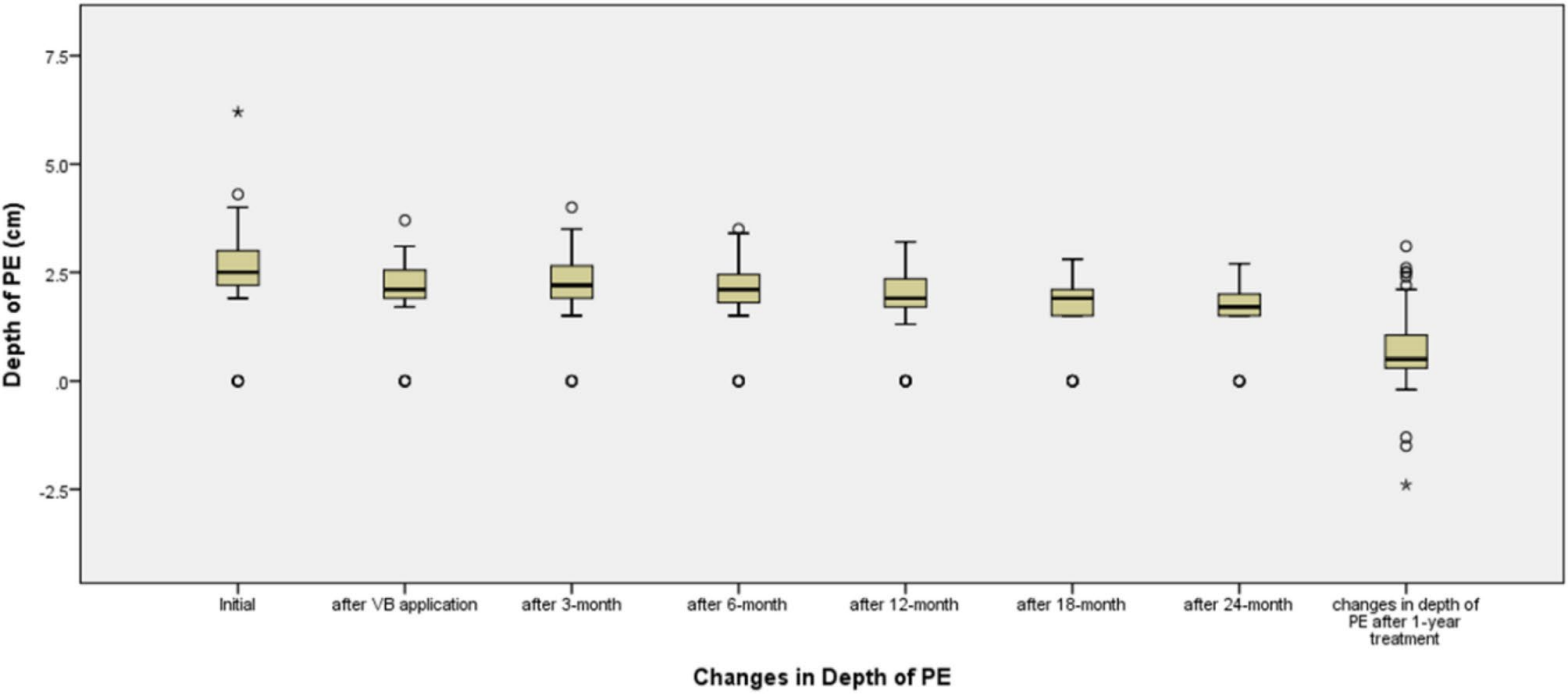
Distribution of depth of pectus excavatum (PE) according to the treatment periods. The initial mean depth was 2.4 ± 1.06 cm (range, 0.0–6.2), and the depth at 5 min after vacuum bell application was 2.0 ± 1.06 cm (range, 0.0–3.7). The actual changes in depth of PE after 1 year of treatment was 0.67 ± 1.06 cm (range, − 2.4–3.10), which was not significantly different between Group 1 and Group 2. The changes in the depth of PE appeared to not be predictive factors of treatment outcomes; the graph could suggest the possible trends in sternal depth after applying a vacuum bell device.

Follow-up BMI had no statistically significant differences between Group1 and Group2 (*p* = 0.390, Table2). BMI of Group2 had significantly increased during treatment periods (*p* = 0.004), and the tendency of improvement in depth of PE seemed to be decreased as the treatment periods elapsed (Fig. 5). This could be indirect evidence for relationship between lower BMI and better treatment expectancy.

Young age at the initiation of treatment was associated with good outcomes. Initiating the therapy before adolescence and age < 12 years demonstrated significantly positive treatment results^12^. Another study reported that age < 18 years was associated with better effects than those after starting treatment at age > 18 years^13^. In our study, age was not significantly associated with improvements in HI (*p* = 0.233, univariate analysis). This was probably due to the age distribution of our patients (mean age, 15.3 ± 6.23 years; 95% confidential interval, 13.78–16.92). Age was not significantly different between the groups. Most of the patients were adolescents; therefore, we could not identify the actual effects of age on efficacy of vacuum bell therapy.

The initial depth of PE in Group 2 was significantly larger than that in Group 1, which was opposite to findings of other studies^3,12,13,15^ that reported that less severe depth in PE was related to excellent outcomes. This might be related to chest wall flexibility; patients with a large depth of PE achieve excellent results if they have good flexibility. The depth of PE measured 1 year after vacuum bell therapy demonstrated no significant differences between the groups (Table 2).

Improvement in the depth of PE demonstrated a flattening tendency as the treatment periods passed more than 1-year in our study (Fig. 5). We believe that this finding could support our study design of comparing pre-treatment variables based on the improvements at 1 year. We evaluated treatment efficacy using chest radiographs and physical examination and would recommend patients to undergo surgery if they had less improvements in HI (usually less than 0.5, according to our findings).

Our study had several limitations. First, we enrolled patients regularly followed according to our treatment strategy. Therefore, we believe that all patients received a relatively homogenous treatment course, which could partly explain the lack of statistical significance. Second, the choice for vacuum bell therapy depended on the patients and not clinical data; patients who were expected to not benefit from non-surgical treatment could be included in our study. Pre-treatment characteristics, such as HI, demonstrated significant differences between the groups. This inclusion bias implies inevitable limitations.

Finally, important variables relating with successful treatment such as duration and time of individual VB usage, compliance and complications were largely dependent on self-reporting data. Currently we had no objective methods for assessing patients’ device applications, there were no other way but to trust their statement. Complications such as skin erythema was described when clinicians identified the lesion during follow-up visit of patients. Skin erosion and chest tightness were reported when patients or their caregivers had insisted. Dependency on subjectivity wound be solved by development of remote equipment based on information technology.

Successful long-term treatment results have been seen with MIRPE for PE. It is important to assure patients that vacuum bell therapy is not a substitute for surgery. MIRPE has resulted in excellent clinical results without fatal complications in a majority of patients^16^. Vacuum bell therapy could offer satisfactory treatment results with few minor complications and less discomfort, and clinicians should not disregard that enormous effort has to be made by the patients and their caregivers to maintain treatment effects of vacuum bell therapy since the hassle of constant devotion often trumps small period of suffering induced by surgery.

In conclusion, expected improvements in HI as well as CI based on pre-treatment chest CT after applying a vacuum bell device could be used in predicting treatment efficacy. Lower BMI was one of positive factors, whereas ages or depth of PE measured 5-min after 30-min application of VB devices, which had been suspected to be involved in optimistic treatment were not. Patients who showed pliability with vacuum bell devices before starting treatment could be identified as suitable candidates.

## Abbreviations

MIRPF: Minimally invasive surgical repair
PE: Pectus excavatum
CT: Computed tomography
HI: Haller index
AI: Asymmetry index
CI: Correction index
BMI: Body mass index
ROC: Receiver-operating characteristic
